# Formation of Slantwise Surface Ripples by Femtosecond Laser Irradiation

**DOI:** 10.3390/nano8070458

**Published:** 2018-06-22

**Authors:** Xin Zheng, Cong Cong, Yuhao Lei, Jianjun Yang, Chunlei Guo

**Affiliations:** 1 The Guo China-US Photonics Laboratory, State Key Laboratory of Applied Optics, Changchun Institute of Optics, Fine Mechanics and Physics, Chinese Academy of Sciences, Changchun 130033, China; lindsax@163.com (X.Z.); leiyuhao93@gmail.com (Y.L.); 2 College of Materials Science and Opto-Electronic Technology, University of Chinese Academy of Science, Beijing 100049, China; 3 School of the Gifted Young, University of Science and Technology of China, Hefei 230026, China; cong2014@mail.ustc.edu.cn; 4 The Institute of Optics, University of Rochester, Rochester, NY 14627, USA

**Keywords:** femtosecond laser, laser-induced periodic surface structures, anomalous slanting ripples, chromium

## Abstract

We report on the formation of slantwise-oriented periodic subwavelength ripple structures on chromium surfaces irradiated by single-beam femtosecond laser pulses at normal incidence. Unexpectedly, the ripples slanted in opposite directions on each side the laser-scanned area, neither perpendicular nor parallel to the laser polarization. The modulation depth was also found to change from one ripple to the next ripple. A theoretical model is provided to explain our observations, and excellent agreement is shown between the simulations and the experimental results. Moreover, the validity of our theory is also confirmed on bulk chromium surfaces. Our study provides insights for better understanding and control of femtosecond laser nanostructuring.

## 1. Introduction

During the last several years, the research of femtosecond laser-induced periodic surface structures (Fs-LIPSSs), or the ripple structures, has attracted tremendous attention because of the abundant scientific issues involved [1,2,3]. Fs-LIPSSs have been studied on a variety of materials, including metals, semiconductors, and dielectrics [4,5,6,7]. It has been found that such microstructures have extensive potential applications, such as magnetic recording media [8], self-cleaning materials [9,10], anti-reflective metals [11], and solar sensors [12].

In general, the distinct characteristics of the ripple structures are closely dependent on the laser parameters. When the linearly polarized single-beam femtosecond laser pulses are used to irradiate materials, the induced ripple structures are either parallel or perpendicular to the direction of the laser polarization [13,14,15,16]. In some cases, however, the ripple structures induced by femtosecond lasers presented an unusual feature of slantwise orientation, which is neither perpendicular nor parallel to the laser polarization direction [17,18,19,20,21,22]. For instance, Qiu et al. [17] reported the slantwise oriented nanogrooves on a ZnO crystal surface with normal incidence of the single-beam femtosecond laser, which tended to be perpendicular to the direction of the laser polarization at the increased scanning speed. By tilting the incident angle of femtosecond laser pulses, Schwarz et al. [19] experimentally observed the slantwise orientation of the ripple structures on fused silica, and the structure orientation changed as a function of the laser incident angle. More recently, our research group generated a series of v-like structures, called a herringbone pattern, on copper [23]. Such anomalous phenomena indicate the physical complexities during the ripple surface structure formation, which is actually significant for femtosecond laser nanoprocessing. Nevertheless, a comprehensive underlying mechanisms of LIPSS orientation is still lacking.

In this work, the formation of slantwise oriented ripple structures is systematically investigated on chromium surfaces by employing single-beam femtosecond laser pulses at normal incidence. First, the ripple structures generated on two lateral edges of the laser-scanned area are seen to have different slantwise orientations with respect to the direction of the laser polarization, and such behaviors occur even when the laser polarization changes. Secondly, based on the measured modulation depth of the ripple structures, we develop a theoretical model to elucidate the underlying mechanisms via the consistent simulations. Finally, additional experiments are performed to confirm the theory.

## 2. Materials and Methods

**Figure 1:**
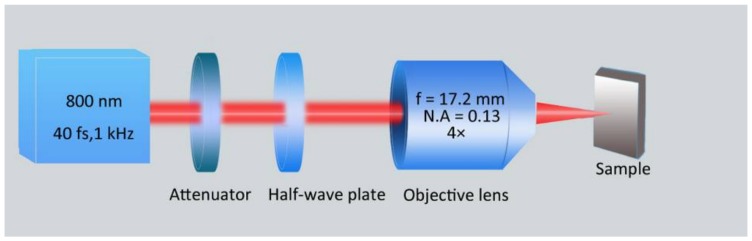
A schematic experimental setup for the formation of slantwise-oriented ripple structures on chromium surface by femtosecond laser irradiation.

As shown by a schematic illustration of the experimental setup in Figure 1, a commercial chirped-pulse-amplification of a Ti:sapphire laser system (Spitfire Ace, Spectra Physics, Santa Clara, CA, USA) was employed as a light source for producing the surface structures, which delivers horizontally polarized femtosecond laser pulse trains with a repetition rate, a central wavelength, and a time duration of 1 kHz, 800 nm, and 40 fs, respectively. The maximum energy of each laser pulse was 7 mJ. Neutral density filters and a half-wave plate were used to control the pulse energy and the direction of the laser polarization, respectively. The laser beam was focused by an objective lens (plan fluorite objective, 4×, *N.A* = 0.13, f = 17.2 mm, Nikon, Tokyo, Japan) at normal incidence. The sample was mounted on a three-dimensional translation stage (ESP301, Newport Inc., Irvine, CA, USA) that could be precisely translated via a custom-made computer program. In order to avoid serious ablation of the material, the sample surface was moved 300 µm away from the focus towards the lens, such that the focus is located inside the sample, resulting in a laser spot radius of ≈39 μm on the sample surface.

In the experiments, 100 nm-thickness chromium (Cr) films deposited on SiO_2_ substrate were chosen as sample materials because of its good physical characteristics, including hardness, corrosion resistance, high melting point, and adhesiveness, which earn many applications in solar absorbers, adhesion layers, micro-electromechanical systems devices, etc. [24,25,26]. Besides, we also used bulk Cr material to carry out experiments. Based on the method of the previous reports [25,27], we experimentally obtained the ablation threshold values of a single laser pulse for the film and bulk of Cr material, which can be given as about 37.5 mJ/cm^2^ and 248 mJ/cm^2^, respectively. All the experimental performances were carried out in ambient air environment. After the laser irradiation, the surface morphologies were investigated using a scanning electron microscope (SEM, Phenom, Eindhoven, Netherlands) and an atomic force microscope (AFM, Bruker, Billerica, MA, USA).

## 3. Results and Discussions

**Figure 2:**
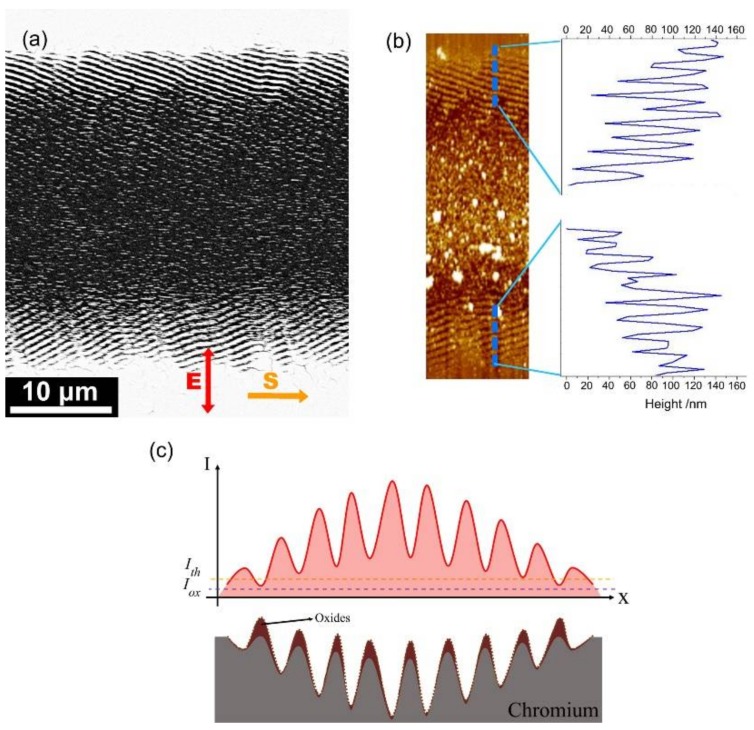
(**a**) SEM image of the ripple structure formation on a Cr film surface irradiated by single-beam femtosecond laser irradiation at the energy fluence of *F* = 56.9 mJ/cm^2^ with the sample scanning speed of *V* = 0.3 mm/s; (**b**) AFM image with the cross-section profiles of the ripple structures formed on both lateral edge regions of the laser-scanned area. Arrows of **S** and **E** represent directions of the sample scanning and the laser polarization, respectively; (**c**) Schematic plots of the periodically distributed intensity distribution on the Cr surface (upper), and its induced ripple structures (bottom), where *I_th_* and *I_ox_* indicate the threshold intensities of the material damage and oxidation processes, respectively. The oxidation layer on the top parts of the surface structures are represented by a purple color.

Figure 2a exhibits the surface morphology of the Cr film irradiated by single-beam femtosecond laser pulses at the fluence of *F* = 56.9 mJ/cm^2^ with the scanning speed of *V* = 0.3 mm/s. Due to the incident Gaussian laser intensity, different surface morphologies could be observed on the area where the laser scanned; substantial ablation damages occurred in the central area, and there were ripple structures on the lateral edges, with a spatial period of approximate 650 nm. In particular, the periodic ripple structures on both edge regions were found to have a slantwise orientation in two different directions. This behavior is in sharp contrast to the previous reports [13,14,15,16], wherein the ripple orientation is usually either perpendicular or parallel to the direction of the incident laser polarization.

To further characterize the features of such slantwise-oriented ripple structures, we employed AFM to measure their modulation depths, and the corresponding results are shown in Figure 2b. Clearly, the measured oscillation curve reveals that the modulation depth of the ripple structures decreases gradually with increasing the distance from the center to the lateral edges of the laser-scanned area, which is due to the spatially inhomogeneous distribution of the laser pulse intensity. On the other hand, the measured peaks suggest that the modulation height of the surface is also varied as a function of the distance from the center of the laser-scanned area. This can be physically understood as follows: The film thickness decreased after irradiation of multiple femtosecond laser pulses, leading to the film thinning at the center of the laser-scanned area with respect to the lateral edge regions. Consequently, the formation of such slantwise oriented periodic ripple structures is in fact based on the gradient variation of the film thicknesses. 

Noticeably, the measured height of the ripple structures was larger than the thickness of the Cr film, which may have been due to material reaction with O_2_ in the ambient atmosphere, leading to oxide formation on the material surface [28]. More specifically, as shown in Figure 2c, there are two physical processes happening in the formation of the laser-induced ripple structures: one is the spatially periodic removal of chromium materials by the modulated laser intensity fringes, and the other is the growth of chromium oxides at the places where the laser intensity is higher than the threshold of oxidation. Usually, the laser damage threshold is larger than that of the oxidation process. Here it should be clear that the periodic femtosecond laser intensity distribution for the ripple structure formation is originated from interference of the light and its excited surface plasmons [29,30,31,32]. Because the two components possess unequal energies, i.e., the energy of the excited surface plasmons is usually smaller than that of the incident laser pulse, their interfering intensity patterns, which had a Gaussian variation profile tend to give a low fringe contrast, or the deconstructive interference fringes can also hold a certain level of the laser energy. Under such circumstances, the material oxidation can take place during the formation of the periodic ripple structures, and the resultant additional oxide layers on the ridge surfaces make the height of the ripple structures become protuberant with respect to the original film thickness.

**Figure 3:**
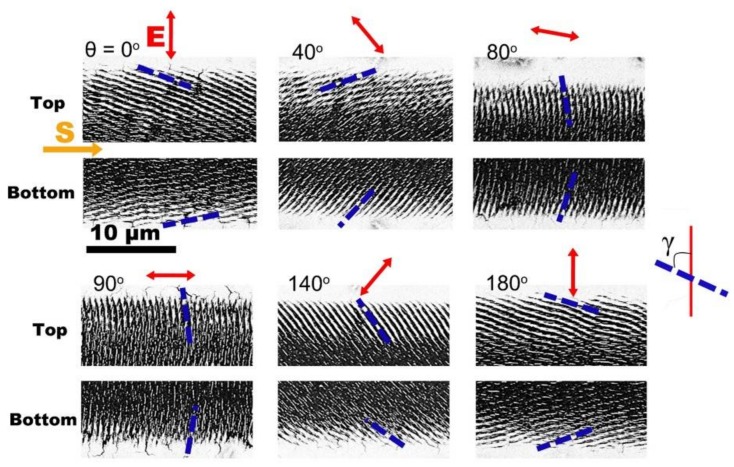
Slantwise-oriented ripple structures on two lateral edge regions (Top and Bottom) of the laser-scanned area on Cr film surfaces by different linear polarizations of single-beam femtosecond laser pulses. The angle *θ* on the upper-left corner of each image represents the direction of the laser polarization. The blue dash lines identify the orientations of the ripple structures. The angle of *γ* indicates an intersection angle between the ripple orientation and the laser polarization direction of *θ* = 0°. The scale bar is applied to all images in this figure.

Inspired by the anomalous phenomenon of the ripple structures with the slantwise orientation, we also performed a series of experiments on Cr films by varying the direction of linear polarization of the femtosecond laser. As shown by the results in Figure 3 (here only the observations on both lateral edges of the laser-scanned area are shown), for the given laser polarization, the slantwise-oriented periodic ripple structures are always produced on both lateral edge regions of the laser-scanned area, being very similar to the observation in Figure 2. Whereas for different laser polarizations, the slantwise degree of the ripple structures is found to change but still neither perpendicular nor parallel to the laser polarization direction. Therefore, we can conclude that the formation of slantwise orientated periodic ripple structures seems to always appear even for different linear polarizations of femtosecond laser pulses.

**Figure 4:**
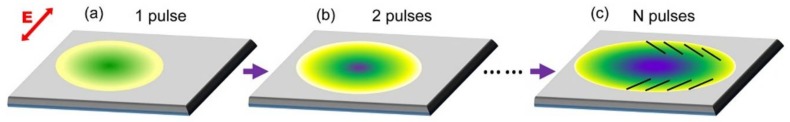
Schematic diagrams of the physical processes for the formation of slantwise-oriented periodic ripple structures on the metal surface. **E** represents the direction of the laser polarization. The different colors represent variations of the modulation depth, which tended to cause the inclined surface within the laser irradiation area.

To elucidate our experimental observations, we proposed the following physical scenario: In our experiments, which are in fact based on multi-pulse femtosecond laser irradiation processes, the pristine surface of the metal film was modified by the preceding incident femtosecond laser pulses, leading to a rough, shallow crater with the modulation depth gradually reducing from the beam center to the peripheral regimes, as shown in Figure 4a, where the inclined surface was created on the laser irradiation area. After that, for the continuous irradiation of the subsequent femtosecond laser pulses, the inclining degree of the laser irradiation surface became pronounced (Figure 4b), and the periodic subwavelength ripple structures were also developed on it, exhibiting the slantwise orientation with respect to the direction of the laser polarization, as shown in Figure 4c. Noticeably, due to the higher intensity distribution on the central region of the laser-scanned area, the formation of the corresponding ripple structures was seriously deteriorated by the accumulating irradiation of subsequent femtosecond laser pulses.

**Figure 5:**
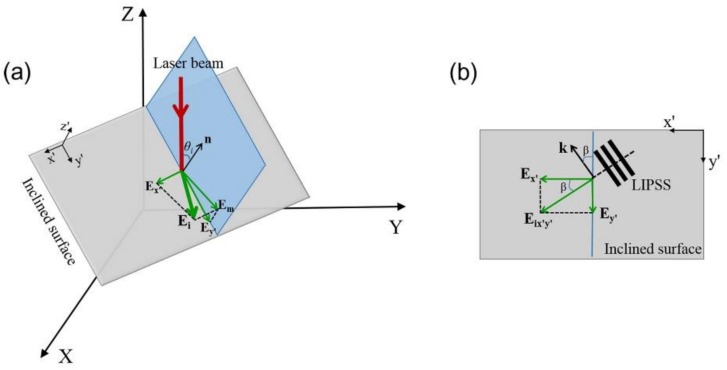
(**a**) A sketch of the laser incidence onto the inclined surface of the material and the decomposition of the electric field **E_i_** onto different planes; (**b**) An effective electric field vector on the x′-y′ plane and its induced periodic ripple structures with the orientation vector **k**.

In fact, the effects of the inclined surface on the formation of slantwise-orientated periodic ripple structures can be theoretically analyzed. According to the previous study of Pham et al. [33], the presence of the inclined surface on the laser spot area can be described by pX+qY+Z=1, within a three-dimensional Cartesian coordinate system X-Y-Z, as shown in Figure 5a. Here *p* and *q* are the geometrical parameters for describing the spatial characteristics of the inclined surface. A normal component of the inclined surface is represented by **n** = (*p*, *q*, 1). Both the propagation direction and the electric field vectors of the incident femtosecond laser are defined as **L_i_** = (0, 0, 1) and **E_i_** = (cos*θ*, sin*θ*, 0), respectively, wherein *θ* is an intersection angle between **E_i_** and the X-axis. As shown in Figure 5a, a plane of the laser incidence (represented by a blue color) is established by the vectors of **n** and **L_i_**, whose intersection angle is defined by *θ_i_*. Moreover, a coordinate system x′-y′-z′ is also built for simplifying the calculation of the electric field on the inclined surface. For the incidence of femtosecond laser on the inclined surface, its electric field vector **E_i_** is divided into two components of **E_x′_** and **E**_m_ through its projection onto the x′-y′ and the incident planes, respectively. On the other hand, the projection of the electric field component **E**_m_ on the x′-y′ plane is indicated by **E_y′_** Finally, in the x′-y′ plane, the two electric field components **E_x′_** and **E_y′_** can be developed into a new vector of **E_x′y′_**, as shown in Figure 5b, with *β* being an intersection angle between **E_x′_** and **E_x′y′_**, which is calculated by the following expression [33]:

(1) $$\;\beta =\arctan\left(\sqrt{\frac{{\left(n\cos\theta_{i}\sqrt{n^{2}-\sin^{2}\theta_{i}+n^{3}-n\sin^{2}\theta_{i}}\right)}^{2}+\kappa^{2}{\left(n^{2}-\sin^{2}\theta_{i}\right)}^{2}}{{\left(n\sqrt{n^{2}-\sin^{2}\theta_{i}}+n^{3}\cos\theta_{i}\right)}^{2}+n^{4}\kappa^{2}\cos^{2}\theta_{i}}}\cot\alpha \right)$$

where $\theta_{i}=\arccos\frac{1}{\sqrt{1+p^{2}+q^{2}}}$ and $\alpha =\arcsin\frac{q\cos\theta -p\sin\theta }{\sqrt{p^{2}+q^{2}}}$.

In Equation (1), *n* and *κ* represent the real and the imaginary parts of the complex refractive index $\tilde{n}$ of the material, respectively. Accordingly, the orientation vector of the ripple structures on the inclined surface, **k**, should be perpendicular to the direction of the electric field **E_x′y′_**, as shown in Figure 5b. When the ripple orientation **k** in the x′-y′-z′ coordinate system is transferred into the X-Y-Z coordinate system, it should be modified into:

(2) $$k=\left(q\sin\beta -\frac{p\cos\beta }{\sqrt{1+p^{2}+q^{2}}},\;-p\sin\beta -\frac{q\cos\beta }{\sqrt{1+p^{2}+q^{2}}},\;\frac{\left(p^{2}+q^{2}\right)\cos\beta }{\sqrt{1+p^{2}+q^{2}}}\right)$$

By considering the actual observation surface happening on the X-Y plane, the orientation vector **k** can be re-written as:

(3) $$k=\left(q\sin\beta -\frac{p\cos\beta }{\sqrt{1+p^{2}+q^{2}}},\;-p\sin\beta -\frac{q\cos\beta }{\sqrt{1+p^{2}+q^{2}}},\;0\right)$$

In the experiments, the orientation vector **k** was obtained by the measurement of angle *γ* shown in Figure 3. Thus, the assumed geometric parameters (*p*, *q*) of the inclined surface could be calculated by the non-linear fitting of Equation (3) with the help of the measured values k=(cosγ,sinγ,0). For example, with the experimentally measured angles of *γ*, the achieved *p* and *q* values were ($p_{\mathrm{top}}=-0.6399$, $q_{\mathrm{top}}=-0.8141$) and ($p_{\mathrm{bottom}}=-0.5077$, $q_{bottom}=0.6690$) for the top and bottom edges of the laser-scanned area, respectively.

**Figure 6:**
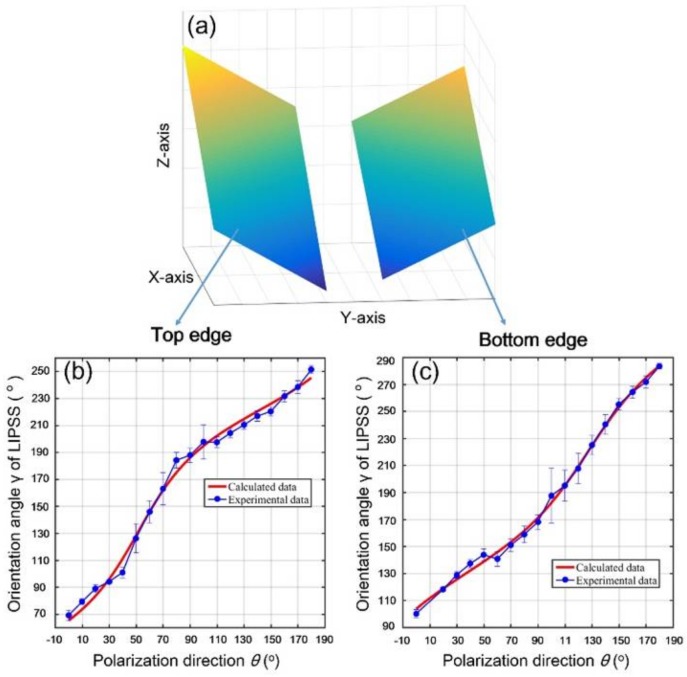
(**a**) Theoretically retrieving the inclined surfaces on the top and bottom edges of the laser-scanned area in the coordinate system of X-Y-Z, with the help of the calculated parameters of ($p_{\mathrm{top}}=-0.6399$, $q_{\mathrm{top}}=-0.8141$) and ($p_{\mathrm{bottom}}=-0.5077$, $q_{\mathrm{bottom}}=0.6690$), respectively; (**b**,**c**) compare the simulation results with the experimental data for the ripple orientation angles on the top and bottom edges of the laser-scanned area, respectively.

Through combing the above calculated *p* and *q* values with the expression of pX+qY+Z=1, we could map three-dimensional profiles of the two inclined surfaces, as shown in Figure 6a, where the left and right surfaces indicate the top and bottom edges of the laser-scanned area, respectively. Evidently, for each inclined surface, the modulation height is varied as a function of the x-y position. In addition, we could also calculate the ripple orientation for the single beam femtosecond laser at different polarization directions. Specifically, because the structure orientation is indicated by the vector k=(cosγ,sinγ,0) where $\cos\gamma =q\sin\beta -\frac{p\cos\beta }{\sqrt{1+p^{2}+q^{2}}}$ and $\sin\gamma =-p\sin\beta -\frac{q\cos\beta }{\sqrt{1+p^{2}+q^{2}}}$, we could obtain *γ* values with the available parameters of *p* and *q*. Therefore, by changing the laser polarization from *θ* = 0° to 180°, the theoretical fitting of the ripple orientation angle *γ* on the top and bottom edges of the laser-scanned area could be obtained, as shown (by red solid curves) in Figure 6b,c, respectively, wherein the experimental data are given (by blue solid circles) with the standard deviation. It is seen clearly that the theory and experiment have good consistency in the two cases. Another feature is that the obtained ripple orientation angle vs the laser polarization direction had nonlinear variations, which was basically due to the polarization dependent optical absorption.

**Figure 7:**
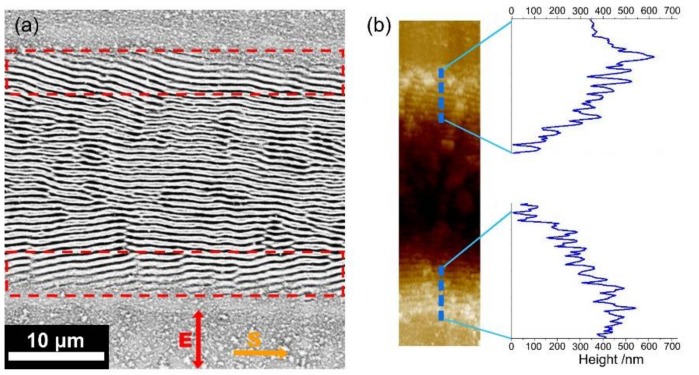
(**a**) SEM image of the ripple structure formation on the surface of Cr bulk material irradiated by single-beam femtosecond laser pulses at the energy fluence of *F* = 40.6 mJ/cm^2^; (**b**) AFM image with the cross-section profiles for the ripple structures on two edge regions of the laser-scanned area.

In order to confirm the above theoretical analyses, we carried out further experiments on the surface of Cr bulk material. As shown in Figure 7a, when the femtosecond laser energy fluence was given at *F* = 40.6 mJ/cm^2^, the subwavelength ripple structures with orientation perpendicular to the laser polarization can still be formed in the central parts of the laser-scanned area. While on both lateral edges (marked by the red dot frames) of the laser-scanned area, the obtained ripple structures exhibit different slantwise orientations. From the corresponding AFM image, as shown in Figure 7b, we can also find that the modulation of the ripple depth is decreased with larger distances from the center of the laser scribed area, which is attributed to the Gaussian beam profile distribution. Clearly, the measured varying tendencies of the ripple depth on the Cr bulk material are very similar to the observations on Cr films.

**Figure 8:**
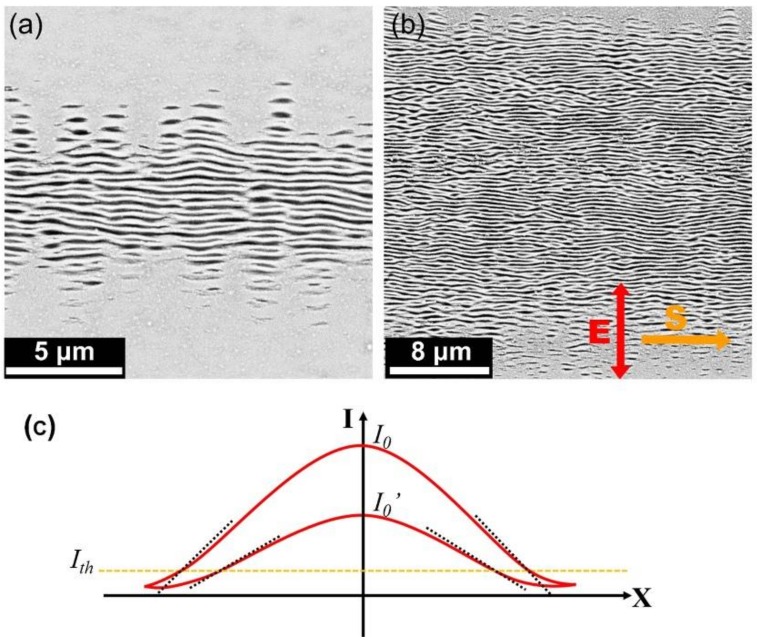
SEM images of the Cr bulk surfaces irradiated by single-beam femtosecond laser pulses with the different energy fluences. (**a**) *F* = 25.7 mJ/cm^2^; (**b**) *F* = 29.6 mJ/cm^2^; (**c**) Variation rates (dot curves) of the laser intensity at the damage threshold (*I_th_*) for two cases of different pulse energy fluences, where *I_0_* and *I_0′_* are the peak intensities of two laser pulses.

On the other hand, when the femtosecond laser energy fluence was decreased to approximately *F* = 25.7 mJ/cm^2^, the ripple structures turned out to be oriented perpendicular to the direction of the laser polarization, especially on both lateral edges of the laser-scanned area, shown in Figure 8a, being similar to many previous reports [13,14,15,16]. As a matter of fact, this situation can be maintained even for the laser energy fluences of about *F* = 29.6 mJ/cm^2^, as shown in Figure 8b, where the ripple-covered region seemed to be enlarged with the orientation still perpendicular to the direction of the laser polarization. Based on the experimental comparisons, it is revealed that the spatial alignment of the ripple structures can be transferred from the slantwise tendency into the direction perpendicular to the laser polarization, if the femtosecond laser energy fluence is weak enough. In other words, the incident larger energy fluence of the Gaussian laser beam was a key factor for the formation of the inclined surface during the multi-pulse laser irradiation, which finally resulted in the slantwise oriented ripple structures on the lateral edges of the laser-scanned area. 

Such a ripple orientation-transferring process can be understood as follows: For a Gaussian laser pulse irradiation on the material with a damage threshold intensity of $I_{th}$, the resultant ablation depth varied as a function of the distance away from the beam center. Therefore, the gradient surfaces were likely to be modified on the ablation edges, with an incline angle *φ* proportional to the variation rate of the laser intensity, i.e., $\varphi \propto \sqrt{\ln\frac{I_{0}}{I_{th}}}$ , where *I*_0_was the peak intensity of the laser pulse. Evidently, with increasing the laser energy fluence, the higher peak intensity could result in a larger incline degree of the ablation surface, as shown in Figure 8c, and the consequent formation of the slantwise oriented ripple structures. Whereas for a femtosecond laser with lower energy fluences, the peak intensity caused a smaller incline degree of the ablation surface, providing negligible influence on orientation of the ripple structures.

## 4. Conclusions

In conclusion, we comprehensively studied the generation of slantwise-oriented subwavelength periodic ripple structures on the surfaces of chromium material by normal incidence of linearly polarized femtosecond laser pulses. Our experimental results on chromium films demonstrated that the ripple structures formed on two lateral edges of the laser-scanned area are slantwise-oriented in two different directions, being neither perpendicular nor parallel to the laser polarization. When the laser polarization direction is changed, the slantwise ripple structures were still observable but with different orientations, AFM measurements suggested that the modulation height of the ripple-covered surface exhibited gradual variations with the distance from the center to the lateral edges, which is due to the inhomogeneous distribution of the Gaussian laser intensity. A physical model was proposed by considering the inclined ablation surfaces after multi-pulse irradiations. The agreement between the simulations and the measured results confirmed the validity of our theory. 

The above mentioned slantwise ripple structures were also generated on bulk Cr surfaces. For the reduced laser fluence, however, the slantwise observations began to transfer into the commonly observed ripple structures with an orientation perpendicular to the direction of the laser polarization, which indicated the negligible influence of the inclined ablation surface. Our investigation provides a comprehensive understanding of femtosecond laser-material interactions, which may help us design and fabricate uniform subwavelength and even nanoscale structures and devices for the future applications.
